# A case report of porcine circovirus 3 (PCV3) reproductive disease in Iberian semi-outdoor reared sows

**DOI:** 10.1186/s40813-024-00407-4

**Published:** 2024-11-21

**Authors:** Àlex Cobos, Marina Sibila, Eva Huerta, Mónica Pérez, Marcial Marcos, Rut Menjón, Marta Jiménez, Laura Gálvez, Joaquim Segalés

**Affiliations:** 1 IRTA, Animal Health, Centre de Recerca en Sanitat Animal (CReSA), Campus de la Universitat Autònoma de Barcelona (UAB), Bellaterra, Catalonia, 08193 Spain; 2 Unitat mixta d’investigació IRTA- UAB en Sanitat Animal, Centre de Recerca en Sanitat Animal (CReSA), Campus de la Universitat Autònoma de Barcelona (UAB), Bellaterra, Catalonia, 08193 Spain; 3 WOAH Collaborating Center for Research and Control of Emerging and Re-Emerging Pig Diseases (IRTA-CReSA), Bellaterra, 08193 Spain; 4 Departament de Sanitat i Anatomia Animals, Facultat de Veterinària, Universitat Autònoma de Barcelona (UAB), Campus de la UAB, Bellaterra, 08193 Spain; 5 MSD Animal Health, Bellaterra, Spain; 6 Montesano Group, Bellaterra, Spain

**Keywords:** Semi-outdoor, Extensive, Porcine circovirus 3 (PCV3), Reproductive disease, PCV3 reproductive disease (PCV3-RD), In situ hybridization (ISH), Periarteritis, Myocarditis

## Abstract

**Background:**

Porcine circovirus 3 (PCV3) is a recently discovered swine pathogen associated with reproductive disease. To date, clinical problems linked to PCV3 have been described in intensive rearing pig farms. The present case describes an Iberian semi-outdoors sow farm affected by PCV3 reproductive disease.

**Case presentation:**

The affected farm was composed of 420 self-replaced Iberian sows, working in 3-week batches (60 sows per batch). The farm was free from porcine reproductive and respiratory syndrome virus (PRRSV) and had been previously affected by porcine circovirus 2 (PCV2) reproductive disease, which was successfully managed through sow vaccination. In spring 2022, reproductive disease was noticed with a high increase in the number of mummified foetuses and stillborn piglets from gilts as the most remarkable finding; multiparous sows were not affected. A first analysis with pooled stillborn tissues ruled out most swine reproductive pathogens and revealed detection of PCV3. To further elucidate PCV3 implication in the reproductive disease, a complete post-mortem examination of stillborn and mummified foetuses from two affected litters was conducted. Pooled tissue samples yielded high PCV3 loads by quantitative PCR. Grossly, one (out of 5) stillborn had an enlarged, flaccid heart. Histopathological evaluation revealed PCV3 lesions consisting of lymphohistiocytic and systemic periarteritis (3/5). The grossly affected heart had lymphohistiocytic myocarditis with fibrosis and lymphohistiocytic endocarditis. By in situ hybridization, high amounts of PCV3 genome were observed within histological lesions. Moreover, immunohistochemistry against PRRSV and PCV2 resulted negative in the same tissues.

**Conclusions:**

This is the first report of PCV3 reproductive disease in a semi-extensive production Iberian pig farm, affecting exclusively gilts. Moreover, this is the first description of grossly apparent myocarditis associated to PCV3 infection. Therefore, PCV3 should be considered within the differential diagnostic list of swine reproductive problems in non-intensive pig rearing production.

## Background

Porcine circovirus 3 (PCV3) was discovered in 2016 in sows affected by reproductive disease and porcine dermatitis and nephropathy syndrome (PDNS)-like lesions [1]. Although initial doubts were raised regarding the PCV3 pathogenic role, its involvement in reproductive disorders was subsequently confirmed. Main associated clinical signs are late abortions, mummified and stillborn foetuses, and weak-born piglets that either die soon after birth or experience a marked decrease in growth rate in later stages [2, 3]. Furthermore, histopathological lesions in cases of PCV3-reproductive disease (PCV3-RD) are also consistent, which include systemic lymphohistiocytic periarteritis and non-suppurative myocarditis [2, 4–6]. The potential of PCV3 to cause transuterine infection and histopathological lesions in piglets has been recently further characterized by means of an experimental infection, which successfully established a link between the etiological agent and the lesions, partially fulfilling Koch’s postulates [7].

Similar to PCV3, porcine circovirus 2 (PCV2) has been known for long to cause reproductive disease, which is characterized by similar clinical signs to those indicated above (abortion, stillbirths, mummifications, preweaning mortalities…) [8]. Interestingly, the pathogenesis of PCV2 reproductive disease (PCV2-RD) depends on the age of foetal infection of tissues, most remarkably cardiomyocytes, which in turn cause a fatal myocarditis [9]. These cardiac lesions can result in a cardiorespiratory insufficiency, grossly apparent as hepatomegaly and anasarca [10–13]. Histologically, the myocardium is infiltrated by lymphocytes and plasma cells, and in most severe cases, necrosis of myocardiocytes and replacement by fibrous tissue appears, which is then referred as necrotizing and/or fibrosing myocarditis [14]. Similarly, foetuses aborted by PCV3-RD have evidence of cardiomyocyte infection and myocarditis [4], and it has been proven experimentally a gestational age-dependent infection of the heart [7].

PCV3-RD has been so far exclusively reported in intensive production herds [4, 5]. Whether PCV3 can produce reproductive disease in extensively reared herds was unknown. Therefore, the purpose of this report is to describe a case of PCV3-RD in an Iberian semi-outdoors herd.

## Case presentation

### Farm description

The case occurred in a semi-outdoors farrow-to-nursery farm, consisting of 420 Iberian sows using a self-replacement policy. The farm was organized in a 3-week batch farrowing system, with 60 sows per batch, and weaning at 4 weeks of age (average 29.1 days). Replacement gilts were introduced during 4 consecutive batches in spring and then 4 more consecutive batches during autumn (overall, 8 out of 17 batches in a year included gilts). Each batch containing replacement stock consisted of 40% gilts and 60% multiparous sows. Gilt management was as follows: the future gilts were housed with their litter mattes and were raised together in the nursery and early fattening stages with the rest of the pigs. At 3 months of age, animals were moved outdoors in fenced areas, and future gilts were allocated in individual fenced areas separated from the rest of fattening pigs. In this area, animals were clinically monitored by visual inspection every day. One month prior to artificial insemination (AI), gilts were relocated indoors within the same building where multiparous sows were also present. At around 40 days after AI, gestation was confirmed using ultrasonography and then multiparous sows and gilts were moved outdoors in separate fields with no contact (in the so-called *soleras*, which are outdoors fenced areas where gilts were allowed to move freely and have access to commercial compound feed and roofed areas). Each group of approximately 20 sows was allocated in a 90 m^2^ space, where they were kept during most of the gestation. One week prior to farrowing, they were reallocated to a maternity room where they farrowed naturally.

The farm had prior history of outbreaks of reproductive disease associated to PCV2 and *Leptospira* spp. (around spring 2021), which were successfully managed with sow vaccination and antibiotic treatment, respectively. At the moment of the present case, the farm’s gilt vaccination program included immunization against *Erysipelothrix rhusiopathiae*, porcine parvovirus (PPV), PCV2, *Leptospira* spp., swine herpesvirus-1, *Brachyspira hyodysenteriae*,* Escherichia coli*, and *Clostridium perfringens* type C. Sows were revaccinated every cycle with the same program. Routinary monitoring of PRRSV was made by means of quantitative PCR (qPCR) in foetuses, stillborn piglets and growing piglets, and ELISA in growing piglets and sows, with continuous negative results. Monitoring of PCV2 was made through qPCR in foetuses, stillborn piglets and growing pigs until 10 weeks of age.

### Clinical description

A reproductive clinical problem was detected during spring 2022, coinciding with the farrowing of a new batch of gilts. Around October 2022 when the next gilt batch was introduced, reproductive problems were observed again, and they were further investigated. All gilts within the four consecutive batches were affected. A minority of the gilts either returned to oestrus or aborted before farrowing date. The remaining ones reached farrowing date, delivering low numbers of alive piglets and an increased number of mummified foetuses and stillborn piglets. The live-born piglets were often weak and failed to grow correctly during the first weeks of life.

During this outbreak of reproductive disease, pooled samples of nine stillborn foetuses (including liver, lung and gastric content) were used to screen reproductive pathogens in an external laboratory by qPCR, including PRRSV, PCV2, PCV3, PPV, *Leptospira* spp., *Chlamydia* spp., and *Brucella* spp. All analyses yielded negative results, except for high PCV3 loads in tissue pools. In consequence, five piglets from two gilt litters were submitted to the *Servei de Diagnòstic en Patologia Veterinaria* (UAB, Barcelona, Spain) to further determine the cause of the reproductive problems and the potential role of PCV3 in them.

### Post-mortem examination

**Fig. 1:**
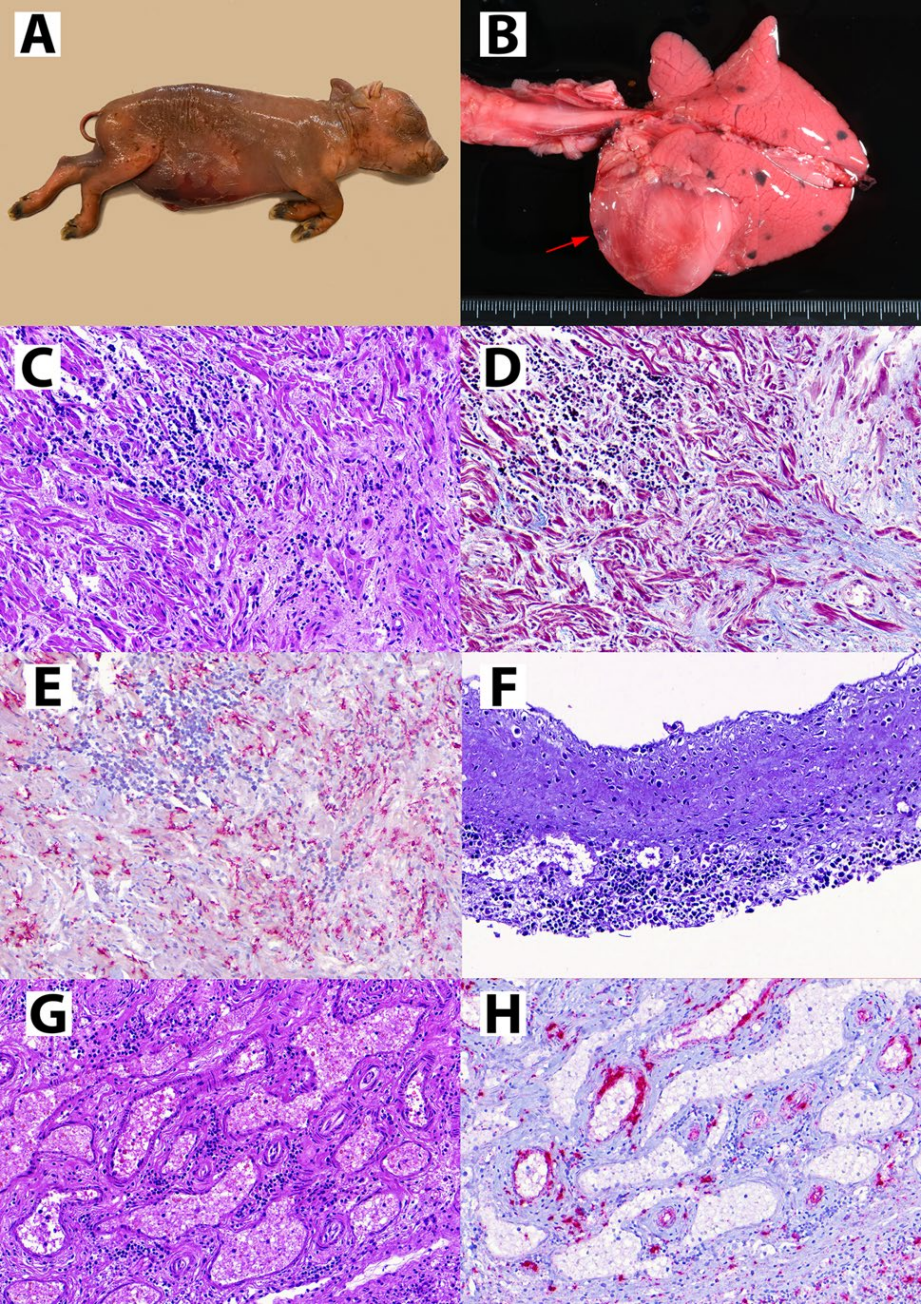
Postmortem examination of stillborn foetus (animal No. 2). (**A**) Severe generalized oedema (anasarca) of the autolytic foetus. (**B**) Grossly evident cardiomyopathy featuring a severe dilation of both ventriculus and depressed reddish areas (red arrow). The lung showed melanosis (black spots), which is a usual finding of Iberian pigs with no disease implications. (**C**) Histopathological evaluation of the heart reveals foci of mononuclear inflammation (top left) and areas devoid of cardiomyocytes and replaced by fibrous tissue (bottom right). (**D**) Masson trichrome stain confirms the presence of collagen fibres (blue colour) in the fibrosed areas. (**E**) ISH against PCV3 reveals moderate amount of PCV3 genome (red staining) within the cardiac tissue. (**F**) Mitral valve, showing subendocardial infiltration of mononuclear cells. (**G**) Mesenteric arteries displaying lymphohistiocytic periarteritis. (**H**) In the same mesenteric arteries, ISH against PCV3 reveals abundant viral genome (red staining) in the arterial walls and -to a lesser extent- within the inflammatory infiltrate

Animals submitted for post-mortem examination consisted of a weak born piglet (animal 1) and a stillborn piglet (animal 2) from one litter, and a weak born piglet (animal 3) and two mummified foetuses (animals 4 and 5) from a second litter. Weight and crown-to-rump length (CRL) of each piglet are detailed in Table 1. Gross lesions were only observed in the stillborn piglet 2, which included generalized oedema (Fig. 1a) affecting the subcutaneous tissue and cavitary effusions (anasarca), as well as cardiomegaly with multifocal dark areas of melanosis (Fig. 1b). Fresh heart, lung, spleen, mesenteric lymph node and brain were collected from all the five piglets and pooled (by animal) for viral load determination through qPCR as described previously [4]. All animals yielded low to high viral loads in pooled tissues (Table 1).

**Table 1:** Summary of histopathological and virological results from necropsied piglets

					Heart		Mesenteric arteries	
Litter	Animal No.	CRL (cm)	Status	Viral load*	Myocarditis	ISH	Periarteritis	ISH
1	1	27	W	1.6 × 10^9^	-	-	+++	+++
	2	25	S	2.9 × 10^9^	++	+++	+++	+++
2	3	20	W	1.6 × 10^4^	-	-	++	+
	4	19	M	7.4 × 10^4^	-	-	-	-
	5	20	M	1.3 × 10^4^	-	-	-	-

*Viral load in a pool of tissues is expressed as PCV3 copies/mL of supernatant of macerated tissue samples. CRL: crown-to-rump length; ISH: in situ hybridization; W: weak born piglet; S: stillborn piglet; M: mummified foetus

For histopathological studies, the following samples were collected from all studied piglets and fixed in 10% neutral-buffered formalin: heart, lung, spleen, mesenteric, tracheobronchial and superficial inguinal lymph nodes, brain, tonsil, liver, kidney, duodenum, jejunum, ileum, colon, placenta and umbilical cord. The tissues were evaluated histopathologically through haematoxilyn-eosin staining, and PCV3-like associated lesions were graded as previously described [15]. Moreover, an in situ hybridization (ISH) targeting the PCV3 rep gene was also performed as previously described [4]. Additional slides were cut and stained using Masson’s trichrome staining to evaluate fibrosis within cardiac tissue. Further slides were obtained to perform immunohistochemistry (IHC) against PCV2 and PRRSV, as previously described [16, 17].

Histopathological evaluation of the heart of animal 2 revealed a moderate lymphohistiocytic myocarditis. Multifocally, loss of cardiomyocytes was observed (Fig. 1c), which was replaced by fibrous tissue as evidenced through Masson’s trichrome stain (Fig. 1d). Abundant PCV3 genome was evidenced through ISH in the myocardium (Fig. 1e). Some mononuclear aggregates could be observed subjacent to the endothelial lining of the endocardium, especially in the heart valves (Fig. 1f).

Lymphohistiocytic arteritis and periarteritis were observed in animals 1, 2 and 3, mainly in the mesenteric arterial plexus and – to a lesser extent – in the renal arteries (Fig. 1g). Score for each lesion is available in Table 1. The same lesions were observed in animal 2 in the placenta and umbilical cord, affecting small and medium caliber arteries but sparing the umbilical artery. All vascular lesions had intense labelling by PCV3 ISH (Fig. 1h). A small degree of interstitial pneumonia was observed in animals 1 and 2, and PCV3 genome was found in alveolar septa in both animals. No lesions were observed in the brain and lymph nodes, although labelling was frequently observed in lymphoid germinal centres and in neuroparenchyma. The other studied tissues were histologically unremarkable. All piglets had negative PCV2 and PRRSV IHC results.

## Discussion

The present case in an Iberian pig farm fulfilled the proposed PCV3-RD diagnostic criteria in three out of five animals, i.e., compatible clinical reproductive signs, vascular histologic lesions, and moderate to high amount of PCV3 genome within the lesions [2]. Importantly, other common swine pathogens (such as PCV2 and PRRSV) potentially linked to reproductive disease [18] were ruled out. Other pathogens such as enteroviruses, teschoviruses and encephalomyocarditis virus were not specifically ruled out; however, to date they have not been reported to cause systemic non-suppurative periarteritis.

In addition, this case provides a previously non-described post-mortem finding in PCV3-RD. One of the stillborn piglets (animal 2) had a grossly identifiable cardiomyopathy, characterized by severe myocarditis with loss of cardiomyocytes and scarring of cardiac tissue with fibrosis. The anasarca observed in the affected animal suggests that cardiovascular failure may play a role in foetal and neonatal death in PCV3-RD. Importantly, the observed myocarditis was similar to that described in PCV2-RD [10, 19–21]. However, subendocardial mononuclear infiltrates had not been reported in previous cases of PCV3-RD although it has been described in a case of PCV2-RD in Norway [22]. Despite these similarities in cardiac lesions between reproductive cases associated to PCV2 and PCV3, a particular difference in the vascular lesions can be noted. Although lymphohistiocytic periarteritis had been occasionally described in PCV2 associated diseases [23], it is a major feature in PCV3-RD cases [4, 5, 7]. Therefore, the thorough histological examination of vascular structures is considered fundamental to support the diagnosis of PCV3-RD together with the detection of the virus in damaged tissues.

In the present case, PCV3-RD affected gilts exclusively. The increased susceptibility of gilts to viral diseases has also been described for PCV2-RD [22], which is probably related to naïve animals being introduced to a herd with endemic circulation of the virus and subsequent infection. In the present case, gilts were initially housed separately from the rest of the herd. As PCV3 likely circulated endemically within the herd, gilts were probably non-exposed during the first months of age and lost maternally derived immunity thereafter, becoming probably naïve unprotected animals. One month before AI, they were transferred to the AI facility in which they were kept in individual cages, but within the same barn than multiparous sows. One month after AI, animals were allocated in groups outdoors (gilts and multiparous sows separately). It is therefore likely that gilts were infected between one month prior and one month after insemination, since it was in this time period where they were kept gilts and multiparous sows in the same environment. It is worth mentioning that PCV3 can be found in semen [24, 25]; however, the role of semen as a potential infectious vehicle for PCV3 is speculative, as for PCV2 the loads in semen of naturally infected boars are not capable of generating PCV2-RD in sows [26]. The moment of infection is presumably a major factor determining the outcome of circoviral infections. Multiple studies already assessed the age-related factors in PCV2 intrauterine infection [9, 27]. Similarly, an experimental PCV3 infection of pregnant gilts revealed that the earlier the infection, the higher viral loads in piglets at farrowing and the more severe PCV3 associated lesions [7]. Although PCV3-RD has been reported in multiple cases affecting intensive farms, this is the first report of PCV3-RD in a semi-outdoor swine herd, which implies that PCV3 may also be a pathogen of concern in facilities with a presumably lower infection pressure. Although speculative, in outdoor housing practices, pigs might have been exposed to wild boar which are well-known natural PCV-3 reservoirs [28] or to infected ticks, which have been suggested to act as vectors [29]. However, this aspect could not be clarified.

The economic burden of PCV3-RD is currently unknown. The most evident impact in PCV3-RD is attributed to direct loss of animals in abortions, mummified and stillborn foetuses. However, affected weak born piglets in this case showed overall poor growth performance in the first weeks of life. This effect has also been reported in previous cases PCV3-RD in the field [5, 6] as well as in piglets from experimentally infected pregnant gilts [7].

PCV3 may produce reproductive losses including mummified and stillborn foetuses. In the present case, mummies had lower viral loads compared to stillborn or weak born piglets, which is similar to previous studies [7]. Moreover, histologic lesions are rarely reported in mummified foetuses due to advanced autolysis. Therefore, for diagnostic considerations, stillborn and/or weak born foetuses are deemed as the best options for diagnostic workup.

Despite PCV3-RD has been known to affect pig herds worldwide for almost a decade, a commercially available vaccine has not been developed. The overall economic impact of PCV3 has not been determined yet. However, it may potentially be prevented through vaccination, as the example of PCV2, which largely prevents PCV2-RD in herds nowadays [26, 30–32]. Some PCV3 vaccine developments are already in place [33].

## Conclusions

This is the first report of PCV3-RD in a semi-outdoor (extensive) swine herd. This case displayed typical PCV3-RD clinical and pathological features, fulfilling PCV3-RD proposed criteria. Moreover, this case offers new undescribed features, such as a grossly visible cardiomyopathy characterized by myocarditis, loss of cardiomyocytes and fibrosis, and subendocardial inflammation.

## Data Availability

No datasets were generated or analysed during the current study.

## Abbreviations

PCV2: Porcine circovirus 2
PCV3: Porcine circovirus 3
PCV2-RD: Porcine circovirus 2 reproductive disease
PCV3-RD: Porcine circovirus 3 reproductive disease
PRRSV: Porcine respiratory and reproductive syndrome virus
PDNS: Porcine dermatitis and nephropathy syndrome
qPCR: Quantitative PCR
ISH: In situ hybridization
AI: Artificial insemination
